# Altered parasympathetic activity during sleep and emotionally arousing wakefulness in frequent nightmare recallers

**DOI:** 10.1007/s00406-023-01573-2

**Published:** 2023-03-02

**Authors:** Vivien Tomacsek, Borbála Blaskovich, Anna Király, Richárd Reichardt, Péter Simor

**Affiliations:** 1 Doctoral School of Psychology, ELTE Eötvös Loránd University, Budapest, Hungary; 2 Institute of Psychology, ELTE Eötvös Loránd University, 46 Izabella Street, Budapest, 1064 Hungary; 3 Institute of Medical Psychology, Faculty of Medicine, Ludwig Maximilian University of Munich, Munich, Germany; 4 National Institute of Locomotor Diseases and Disabilities, Budapest, Hungary; 5 Institute of Education and Psychology at Szombathely, ELTE Eötvös Loránd University, Budapest, Hungary; 6 UR2NF, Neuropsychology and Functional Neuroimaging Research Unit at CRCN-Center for Research in Cognition and Neurosciences and UNI-ULB Neurosciences Institute, Université Libre de Bruxelles (ULB), Brussels, Belgium

**Keywords:** Sleep, Nightmare, Heart rate, Heart rate variability, Dreaming

## Abstract

Nightmare disorder is characterized by dysfunctional emotion regulation and poor subjective sleep quality reflected in pathophysiological features such as abnormal arousal processes and sympathetic influences. Dysfunctional parasympathetic regulation, especially before and during rapid eye movement (REM) phases, is assumed to alter heart rate (HR) and its variability (HRV) of frequent nightmare recallers (NM). We hypothesized that cardiac variability is attenuated in NMs as opposed to healthy controls (CTL) during sleep, pre-sleep wakefulness and under an emotion-evoking picture-rating task. Based on the polysomnographic recordings of 24 NM and 30 CTL participants, we examined HRV during pre-REM, REM, post-REM and slow wave sleep, separately. Additionally, electrocardiographic recordings of resting state before sleep onset and under an emotionally challenging picture-rating task were also analyzed. Applying repeated measures analysis of variance (rmANOVA), a significant difference was found in the HR of NMs and CTLs during nocturnal segments but not during resting wakefulness, suggesting autonomic dysregulation, specifically during sleep in NMs. As opposed to the HR, the HRV values were not significantly different in the rmANOVA in the two groups, implying that the extent of parasympathetic dysregulation on a trait level might depend on the severeness of dysphoric dreaming. Nonetheless, in the group comparisons, the NM group showed increased HR and reduced HRV during the emotion-evoking picture-rating task, which aimed to model the nightmare experience in the daytime, indicating disrupted emotion regulation in NMs under acute distress. In conclusion, trait-like autonomic changes during sleep and state-like autonomic responses to emotion-evoking pictures indicate parasympathetic dysregulation in NMs.

**Supplementary Information:**

The online version contains supplementary material available at 10.1007/s00406-023-01573-2.

## Introduction

Nightmares are vivid, dysphoric dreams that usually result in abrupt awakenings. They mostly occur during late-night rapid-eye-movement (REM) or Non-REM (NREM) Stage 2 sleep [1]. Upon awakening, full alertness is regained with a clear recall of mentation, which has a substantial negative effect on sleep quality, and could also influence one’s overall psychological well-being [2, 3]. The prevalence of weekly nightmares is 2–6% in the general population, which might be an indicator of further psychopathological processes; nevertheless, a greater quantity of nightmares can be observed in childhood and less in the elderly, while female nightmare reports are more frequent than male ones [2, 4–6]. Dysphoric dreaming is even more common in psychiatric populations [7], and indicates the severity of psychopathology [8]. Nightmares due to trauma exposure are considered distinct from idiopathic nightmares, which are clearly not related to specific traumatic experiences and their etiology remains unknown [2].

There have been several theoretical considerations that make an attempt to elucidate the pathogenesis of nightmares. In their model, Nielsen and Levin interpret it on neurophysiological and cognitive levels, pointing out the strong association between waking psychological functioning and nocturnal disturbances [6]. The authors propose that one of the functions of (dysphoric) dreaming is the extinction of fear-related memories; in nightmare disorder, however, the regulation of fear memories is dysfunctional and not efficient enough to downregulate the emotional intensity of negative dream experiences. State-like factors of ‘affect load’ might increase the incidences of dysphoric dreams, while ‘affect distress’, referring to the predisposition of being reactive to emotional stimuli, escalates nightmarish experiences and contributes to the clinical severity of frequent nightmares [6, 9, 10]. Nielsen’s more recent theory regarding idiopathic nightmares stipulates that adverse events in the infantile amnesia period may alter the neural network responsible for fear extinction, leading to fear sensitivity [11]. Childhood adversity, which is not necessarily a traumatic experience, is also one of the factors mentioned by Gieselmann and colleagues as antecedents of nightmares [12]. Merging the already existing literature on the etiology of nightmares, Ellis based on Porges’ polyvagal theory [13] points out that the autonomic nervous system (ANS) not only conveys a response to nightmares, but it also contributes to their cause through reactions to threat and heightened sensitivity [14].

Uncovering the pathophysiological processes of frequent nightmares is of utmost importance to fully understand this disorder. The sleep of participants with chronic nightmares is fragmented due to awakenings and microarousals, and is characterized by a decrease in low frequency and an increase in high-frequency electroencephalographic (EEG) spectral power indicating disrupted sleep regulation, especially during NREM sleep [15–17]. Other signs of abnormal physiological activity are also observed in frequent nightmares, such as dyspnea, sweating or periodic leg movements in REM sleep [16]. Sleep microstructural changes (such as microarousals) during NREM to REM transitions [18–21] are amplified in frequent nightmare recallers (NM) [15]. In general, NREM to REM transitions (pre-REM segments) appear to be more fragile, with intensified cardiac activity and microarousals [22–24], while REM to NREM changes (post-REM phases) are more stable periods, when protection from arousals is more efficient [18, 24].

The assessment of cardiac activity as a physiological correlate of autonomic activation has become widespread in both emotion and sleep research [25, 26]. Increased heart rate (HR) and decreased heart rate variability (HRV) indicate the activation of the sympathetic tone and attenuated parasympathetic regulation in the ANS. HRV can be applied to trace alterations in emotional states [27], such as changes in emotional valence and intensity [28]. For instance, viewing pictures with negative valence reduced HRV [29], whereas higher HRV was linked to well-being and emotion regulation [30]. Previous research pointed to altered frontolimbic activity in NMs while viewing emotionally negative pictures [31], while others reported elevated subjective arousal to emotional pictures in NMs regardless of valence [32]. These papers, however, did not consider the evaluation of ANS activity.

Although parasympathetic activity (PA) is generally more dominant during supine rest and nocturnal sleep compared to active wakefulness, reflecting a diurnal pattern as well [33], ANS activity is also under ultradian regulation showing increased PA in slow wave sleep [34–37] and reduced PA in REM sleep [38]. Although a number of studies have already observed decreased PA in posttraumatic stress disorder (PTSD) [39–45], the existing reports on non-traumatic nightmares and HRV are inconclusive. In laboratory settings, Nielsen and colleagues found no difference in HRV across NMs and healthy controls (CTLs) during pre-sleep wakefulness but observed significantly lower HRV in NMs during the recovery night after REM sleep deprivation, especially during REM phases [46]. Simor and colleagues reported reduced HRV in pre-REM phases and non-transitory NREM periods in NMs compared to CTLs [24]. More recently, in an ambulatory study Paul and colleagues observed attenuated HRV in NMs only after nightmares during REM sleep, but did not find any group differences in ANS activation in REM sleep irrespective of dream recall [47].

Some methodological aspects of HRV, however, have remained unaddressed. Certain frequency components of the HRV were originally thought to be markers of the sympathetic activity (i.e. the low frequency component) [48–50] and the so-called sympathovagal balance (i.e. the ratio of the high and low frequency components) [51]. These concepts have been widely challenged, and it has been proposed that by means of HRV measures, only the parasympathetic tone of the ANS can be consistently assessed [48, 52, 53]. Therefore, here we focus solely on parasympathetic changes.

Taking into account both the theory of Nielsen and Levin concerning nightmare production, and the available data on the autonomic functioning of NMs, it may be presumed that the enhanced emotional reactivity and the failures in fear-response elimination that accompany disturbed dreaming could impact the physiological functioning as well, potentially decreasing the sufficient regulation of the parasympathetic nervous system in frequent nightmare recallers [6]. In line with this model, we might assume NMs to have the disposition to demonstrate altered autonomic responses due to the ‘affect distress’ regardless of current negative life-events (e.g. during sleep, irrespective of dream presence or quality), while the phenomenon might also be intensified by acute stressful experiences (‘affect load’) (e.g. due to negative mood induction). Moreover, adverse experiences in childhood are also believed to impact the ANS and emotion regulation capacity negatively, leading to susceptibility to threat and stressors and ANS dysregulation, as highlighted by the polyvagal theory [12–14].

The aim of the present study is to explore the PA in idiopathic NMs during wakefulness and sleep. We hypothesize that NMs exhibit less PA during sleep and a daytime emotionally challenging task (i.e. rating emotion-evoking pictures), which will be reflected in accelerated HR and attenuated HRV as opposed to CTLs.

## Methods

### Participants

Participants were either students of two Hungarian universities or recruited through social media. The eligibility of any potential candidate was assessed with a set of online questionnaires, which was followed by an interview to verify the frequency of nightmares and to exclude participants with trauma-related recurring nightmares. The selection of the participants was based on their scores in three questionnaires: Dream Recall Frequency [54], Nightmare Frequency and Bad Dream Frequency (i.e. nightmares without awakening) [55]. Two groups of subjects were selected, who both reported recalling their dreams at least once every 2 weeks. Those having at least one nightmare or bad dream per week were assigned to the NM group, while those reporting nightmares or bad dreams less than every two or three months were the CTL group. None of the subjects reported any prior neurological, psychiatric or sleep disorders, trauma- or temporary stress-related nightmares, chronic diseases or regular medication (except contraceptives) [15, 22]. In order to quantify nightmare-related emotional distress, the participants also filled in the Van Dream Anxiety Scale [56]; and to assess emotional distress more generally, the Beck’s Depression Inventory [57], and the items of the State-Trait Anxiety Inventory [58] concerning trait anxiety were also completed. In Table S1, additional information is provided on these constructs. The recruitment, as well as the subjective measures, are described in detail in our previous studies [15, 22].

62 participants (28 NMs and 34 CTLs) were found eligible for the study. However, one participant left the experiment after the first night and the data of seven additional subjects were excluded from the final dataset due to technical problems or comorbidities. Eventually, the data of 24 NMs without any comorbidities (*M*_age_ = 23.125; SD_age_ = 3.327) and 30 CTLs (*M*_age_ = 22.1; SD_age_ = 3.487) (see Table 1 for descriptive statistics) was analyzed. The experiment consisted of two main phases (see Procedure). In the nocturnal analysis, the data of 52 subjects was included (23 NMs and 29 CTLs), while in the picture-rating task, 49 participants’ data was used (23 NMs and 26 CTLs). The datasets of the two analyses largely overlap.

**Table 1:** Descriptive statistics of study participants

Groups	NM (*N* = 24)		CTL (*N* = 30)		Group comparisons
					Test statistics (*p* value)
Gender	M (*N* = 9)	F (*N* = 15)	M (*N* = 11)	F (*N* = 19)	0.004 (0.95)^a^
Mean age (SD)	23.1 (2.9)	23.1 (3.6)	23.3 (5.1)	21.4 (2.0)	277.5 (0.147)^b^
Mean height in centimeter (SD)	183.8 (7.6)	169 (4.2)	180.4 (6.5)	166.7 (7.8)	− 1.077 (0.288)^c^
Mean weight in kilogram (SD)	87.3 (17.7)	58.25 (7.7)	80.6 (22.9)	60.7 (11)	160.5 (0.731)^b^
Mean BMI (SD)	25.7 (3.5)	20.6 (3)	24.5 (5.3)	21.8 (3.3)	177.5 (0.893)^b^

*NM* frequent nightmare recaller participants; *CTL* control participants; *M* male; *F* female; *N* number of participants; *SD* standard deviation; *BMI* body mass index

^a^Indicates *χ*^2^ test

^b^Indicates Mann–Whitney test

^c^Indicates Student’s *t* test

University students were compensated with partial credit points for participating in the experiment, while other subjects received monetary compensation (approximately 45€ in Hungarian forints). The study protocol was approved by the United Ethical Review Committee for Research in Psychology, Hungary (EBKEB 2016/077), and written informed consents were obtained.

### Procedure

The participants spent two consecutive nights in the sleep laboratory and had been instructed beforehand not to nap during the day or drink any caffeinated or alcoholic beverages after 2 pm. Their arrival at the laboratory was between 09:00 and 10:30 pm. Bedtimes were scheduled after the preparation of the polysomnography, between 10:00 and 11:30 pm, adjusted to the preference of each participant. The subjects slept at least 7 h and were awakened between 07:00 and 08:00 am, accordingly. A similar schedule was repeated on the second night with an additional task before the polysomnographic electrode placement. In this task, participants were shown and asked to provide arousal and valence ratings on a set of negative and neutral International Affective Picture System (IAPS) pictures [59], while their heart rate and skin conductance were detected. Since the first night served as a habituation night, the polysomnographic data recorded on the second night is analyzed here.

### Measures

#### Polysomnography

All participants were fitted with 17 EEG electrodes (F7, F8, F3, F4, Fz, T3, T4, C3, C4, Cz, T5, T6, P3, P4, Pz, O1, and O2) according to the 10–20 electrode placement system [60], referred to the mathematically linked mastoid (A1 and A2) electrodes. We used bipolar electromyography (EMG) placed on the chin, as well as electrooculography (EOG) and electrocardiography (ECG). Gold coated Ag/AgCl EEG cup electrodes were fixed with EC2 Grass Electrode Cream (Grass Technologies, Natus Manufacturing Ltd., Galway, Ireland). Data were recorded with Micromed SD LTM 32 Bs (Micromed S.p.A., Mogliano Veneto, Italy) and SystemPLUS 1.02.1098 software (Micromed Srl, Roma, Italy). Impedances were below 8 kΩ. Signals were collected, pre-filtered (0.33–1500 Hz; 40 dB/decade anti-aliasing hardware input filter) and digitized with 16-bit resolution. After that, the pre-filtered, amplified and digitized signal was downsampled to 512 Hz.

#### Picture-rating task

Participants were shown 20 pictures selected from the IAPS [59] in three repetitive blocks. The blocks consisted of 10 negatively valenced and highly arousing, and 10 neutral (and moderately arousing) pictures. Both the negative and the neutral pictures belonged to different categories and were selected on the basis of the standardized arousal and valence scores provided by the IAPS database [61] as well as our own pilot experiments.

First, the participants viewed a black screen for 1 min and were asked to stay calm and prepare for the upcoming pictures. Then, they were familiarized with the task by viewing and rating neutral pictures (fillers), which were not included in the analyses. After that, the experimental phase commenced, and in each block the same pictures were presented in a randomized order. Each picture was preceded by a fixation cross presented for a period varying randomly between 8 and 12 s; then the picture appeared for 10 s. After each, participants were asked to rate the arousal and valence values of the current picture in accordance with the scoring system of the IAPS [61]. The task was presented and responses were recorded on a laptop (1920 × 1080 pixels) using OpenSesame (Version 3.1.9.) [62]. Along with subjective ratings, physiological data (heart rate and skin conductance) was assessed during the task. Here we focus only on the ECG recordings, as that was also measured during sleep. The ECG signal was recorded with the NeXus recording system (NeXus Wireless Physiological Monitoring and Feedback: NeXus-10 Mark II, Version 1.02; BioTrace + Software for NeXus-10 Version: V201581; Mind Media BV, Herten, the Netherlands) and electrodes were placed at the upper right and left, and below the lower left sternal border.

### Data analysis

Sleep stages were manually scored according to the standardized criteria of the American Academy of Sleep Medicine [63] by experts trained in sleep research.

For the nocturnal heart rate analysis, 10-min-long ECG recordings were extracted from four sleep stages: (1) stable NREM (i.e. NREM Stages 2 and 3), (2) pre-REM (i.e. 10 min directly preceding a REM phase), (3) REM and (4) post-REM (i.e. 10 min directly following a REM phase); and the entire period spent awake before sleep onset, using MATLAB (version 8.3.0.532, 2014a, The MathWorks, Inc., Natick, MA) software. As for the picture-rating task, the subjective ratings of the pictures in valence and arousal in the experimental phase were analyzed, as well as their ECG recordings during this entire phase.

HRV analysis was carried out with Artiifact 2.13 software [64]. The peaks of R-waves were detected automatically and screened visually to correct missed or misplaced R-peaks manually. Thenceforward, artifacts were detected and processed in the segments with cubic spline interpolation and Bernston algorithm [65], but to preserve the validity of the analysis, the minimum length of the selected segments was set to 2 min. For the evaluation of the variations of HR, time and frequency domain indices can be applied [66, 67]. In the present study, the mean HR, the root mean square of successive differences (RMSSD) and the high-frequency component (HF) were computed. Their selection was based on their reliability and validity as markers of PA [48, 68]. In the nocturnal segments, the values of the three measures were averaged within each sleep stage for every participant. As for the pre-sleep wakefulness and the experimental phase of the picture-rating task, the cardiac activity during these entire periods was assessed.

The evaluation of the daytime HRV in emotionally challenging conditions apart from the nocturnal segments enabled us to use the cardiac responses during the picture-rating task as a model of emotional reactivity while experiencing a nightmare, since having a nightmare in a sleep laboratory has been found to be rare [6]. The presentation of both negative and neutral pictures served as a model of a dysphoric dream experience, which not only contains negative, but also less arousing emotions [69]. The inclusion of neutral pictures aimed to prevent a potential habituation to the negative ones. As for the subjective ratings of the pictures, the mean scores were computed in valence and arousal for each participant.

In the present analysis, the raw HRV scores were taken into account, and when non-parametric tests were not available, the RMSSD and HF values were transformed using the natural logarithm in order to approximate normal distribution. While in the case of frequency components of the HRV, the recommendation is to report both absolute and relative power values [66, 70], recent findings suggest that the normalized units convey mathematically redundant results and might lead to false interpretations [71, 72].

Statistical analyses were carried out with JASP (Version 0.13.1.0, Team JASP, 2018, Amsterdam) and IBM SPSS Statistics (Version 28). Alpha level was set to 0.05; in the case of multiple comparisons, however, adjusted *p*-values were applied. Skewness and kurtosis of data distribution and the Shapiro−Wilk tests were used to assess the normal distribution of each variable. Differences in HRV measures across wakefulness, pre-REM, REM, post-REM and stable NREM periods between the NM and CTL groups were examined with a repeated measures univariate analysis of variance (rmANOVA) model for each measure. We tested 5 × 2 models including the following independent factors: Phase (wakefulness, pre-REM, REM, post-REM, stable NREM) as a within-subject repeated measures factor and Group (NMs, CTLs) as a between-subject factor. We also tested 2 × 2 models with the following independent variables: Phase (pre-REM, post-REM) as a within-subject repeated measures factor and Group (NMs, CTLs) as a between-subject factor. If the Mauchly test indicated that the sphericity was violated, Greenhouse–Geisser correction was applied. Uncorrected degrees of freedom, *p* values (adjusted, in case of multiple comparisons) and partial eta-squared (η_p_^2^) as a measure of effect size are reported. Post hoc group differences and the differences in subjective ratings and cardiac responses during the emotion-evoking task between the NMs and CTLs were evaluated with Student’s *t* test (in case of normal distribution), Welch-test (in case of unequal variance) or bootstrapping (if normal distribution was not fulfilled) based on 1000 bootstrap samples. In these cases, effect sizes are reported in Cohen's *d* value. In these group comparisons, the HR or HRV values, or the subjective ratings were the dependent variables, and the group membership (NM or CTL) was the independent variable. Additional correlation analyses were conducted with bootstrapping based on 1000 bootstrap samples applying Pearson’s correlation, reporting the correlation coefficient *r* as effect size.

## Results

### Nocturnal segments

**Fig. 1:**
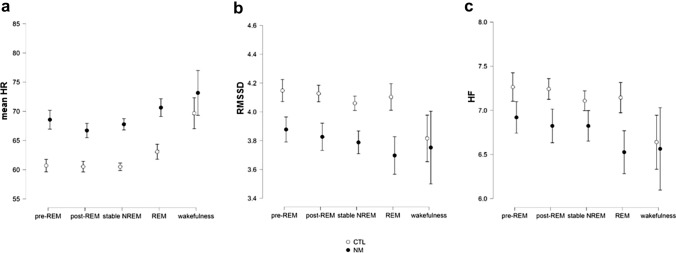
HR (**a**) and HRV (**b**, **c**) measures of the NM and CTL groups within the examined sleep periods. *NM* nightmare group; *CTL* control group; *HR* heart rate. Error bars show confidence intervals (95%)

The mean HR of all the participants was within normal ranges (sleep: 45.528–85.551; pre-sleep wakefulness: 46.021–100.713). In this index, we found significant main effects of Phase (*F*(1.397) = 28.138; *p* < 0.001; *η*^2^_p_ = 0.38) and Group (*F*(1) = 7.339; *p* = 0.009; *η*^2^_p_ = 0.138) (Fig. 1a); however, the Group × Phase interaction was nonsignificant (*F*(1.397) = 2.192; *p* = 0.135; *η*^2^_p_ = 0.045).

The post hoc comparisons showed that overall, the NM group had higher mean HR than the CTLs (*d* = − 0.391; MD = − 6.473), and there were remarkable differences between certain sleep stages (see Fig. 1a). As Table 2 demonstrates, the HR was significantly higher in REM and wakefulness than in NREM periods (i.e. pre-REM, post-REM and stable NREM).

**Table 2:** Significant pairwise comparisons of heart rate in different sleep and vigilant states

Sleep phases	*t *test	*p*_Holm_	Cohen’s *d*
Pre-REM vs. REM	− 2.6	0.04	− 0.375
Pre-REM vs. wakefulness	− 7.944	< 0.001	− 1.147
Post-REM vs. REM	− 3.795	0.001	− 0.548
Post-REM vs. wakefulness	− 9.139	< 0.001	− 1.319
Stable NREM vs. REM	− 3.185	0.008	− 0.46
Stable NREM vs. wakefulness	− 8.529	< 0.001	− 1.231
REM vs. wakefulness	− 5.344	< 0.001	− 0.771

*p* values are adjusted with Holm method for comparing multiple (10) variables

Regarding the RMSSD, neither the main effect of Group (*F*(1) = 3.718; *p* = 0.06; *η*^2^_p_ = 0.075) nor the Group × Phase interaction (*F*(1.646) = 2.403; *p* = 0.107; *η*^2^_p_ = 0.050) were significant (Fig. 1b). Moreover, no significant results were observed in the main effect of Group or the Group × Phase interaction of the HF either (*F*(1) = 1.693; *p* = 0.2; *η*^2^_p_ = 0.035; and *F*(1.820) = 1.637; *p* = 0.203; *η*^2^_p_ = 0.034, respectively) (Fig. 1c). However, similarly to the mean HR, the main effects of Phase were found significant in both the RMSSD and the HF (*F*(1.646) = 4.777; *p* = 0.016; *η*^2^_p_ = 0.094; and *F*(1.820) = 3.901; *p* = 0.004; *η*^2^_p_ = 0.122, respectively; see Fig. 1b and /c).

Regarding the group comparisons, NMs had elevated heart rate during all sleep states, but not during pre-sleep resting wakefulness (Table 3).

**Table 3:** Group comparisons of heart rate in sleep periods and resting pre-sleep wakefulness

Sleep phases	Test statistic	df	*p*	MD	Cohen’s *d*
Pre-REM	− 2.734	50.000	0.018*	− 6.756	− 0.763
Post-REM	− 2.202	50.000	0.040*	− 5.291	− 0.615
Stable NREM	− 2.630	50.000	0.018*	− 6.419	− 0.734
REM^+^	− 2.714	38.377	0.018*	− 6.245	− 0.772
Wakefulness	− 1.030	46.000	0.308	− 3.485	− 0.302

Test statistic is the *t* value of either Student’s or Welch’s *t* test (^+^ indicates the inequality of variance, hence Welch’s *t*). * indicates significant results. FDR-adjusted *p* values are reported

*Df* degrees of freedom; *MD* mean difference

**Fig. 2:**
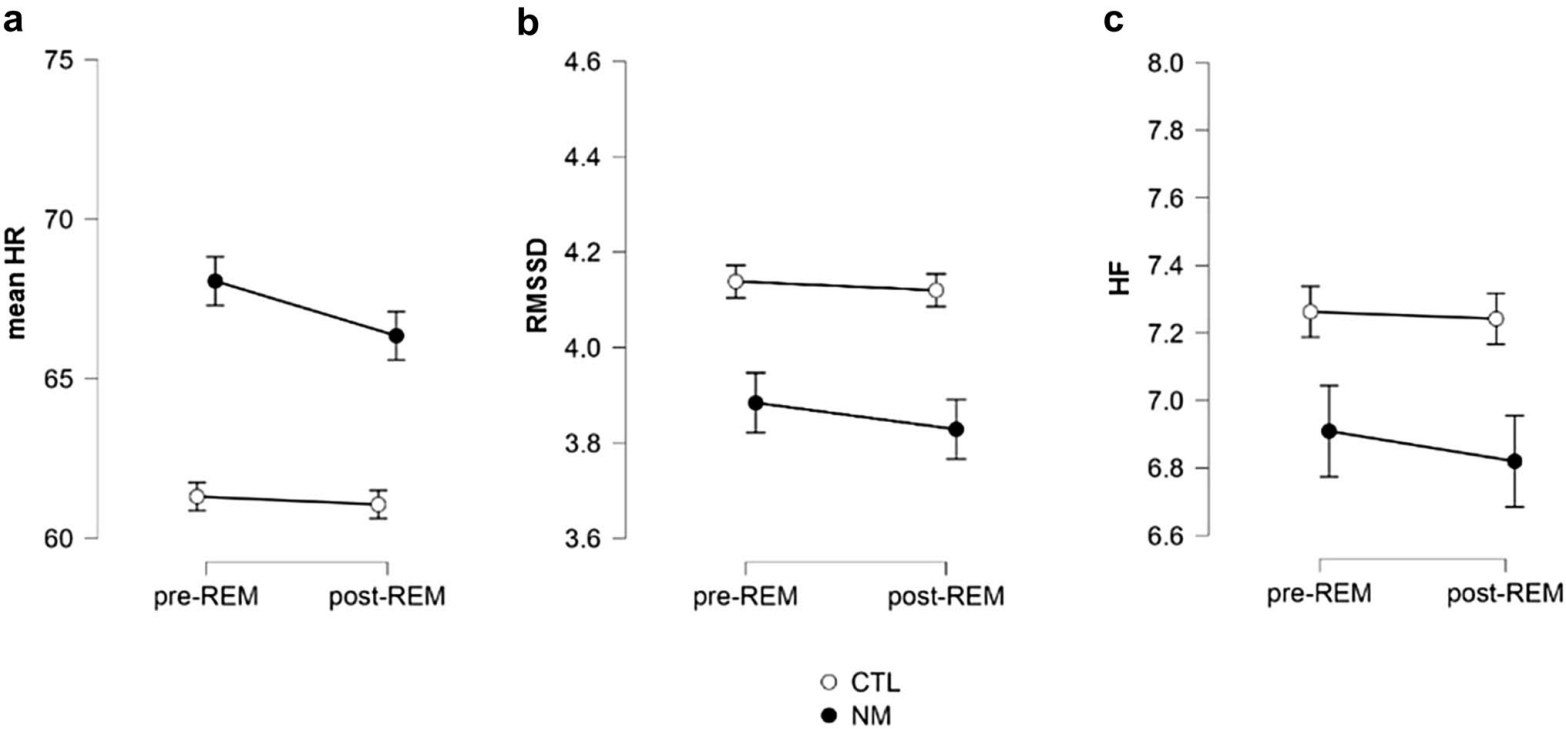
Mean heart rate (**a**) and heart rate variability (**b** and **c**) during pre- and post-REM periods in the NM and CTL group. *NM* nightmare group; *CTL* control group; *HR* heart rate; *RMSSD* root mean square of successive differences; *HF* high frequency component of the HRV. Error bars show confidence intervals (95%)

**Fig. 3:**
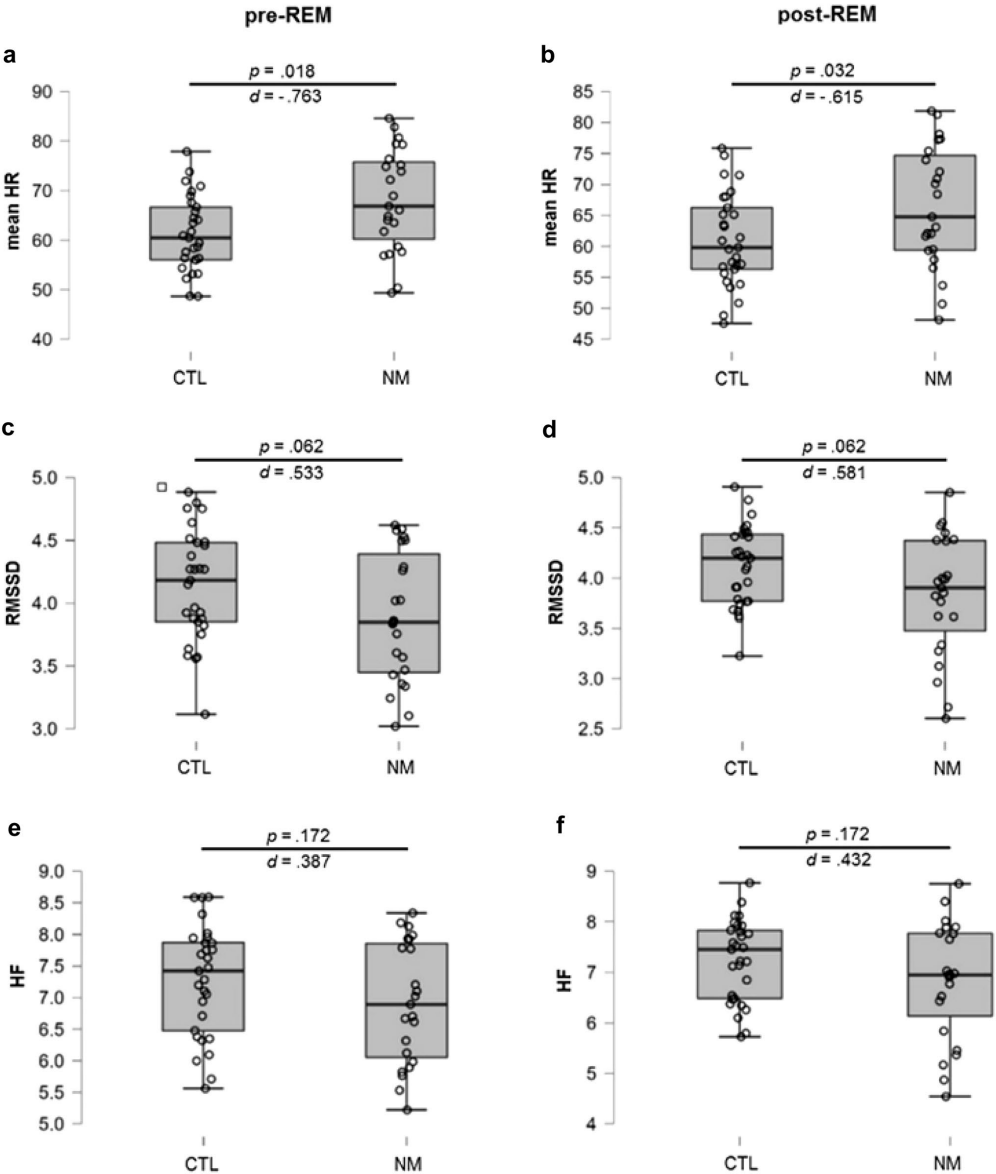
Mean heart rate (**a**, **b**) and heart rate variability (**c**–**f**) during pre- and post-REM periods in the NM and CTL groups. *NM* nightmare group; *CTL* control group; *HR* heart rate; *RMSSD* root mean square of successive differences; *HF* high frequency component of the HRV. FDR-adjusted *p* values are reported. Error bars show confidence intervals (95%)

To replicate Simor and colleagues’ previous results [24], further exploratory analyses were conducted, where pre- to post-REM changes were compared in mean HR, RMSSD and HF between groups. In HR, the main effect of Group was significant (*F*(1) = 6.195; *p* = 0.016; *η*^2^_p_ = 0.110), indicating increased HR in NMs compared to controls. In addition, the main effect of Phase (*F*(1) = 11.752; *p* < 0.001; *η*^2^_p_ = 0.190) and the Phase × Group interaction (*F*(1) = 6.580; *p* = 0.013; *η*^2^_p_ = 0.116) were also significant. The post hoc analysis revealed that the NM group showed significantly higher mean HR (*t* = − 2.773; *p*_*Holm*_ = 0.031; MD = − 6.756) than the CTLs, specifically in pre-REM phases, whereas group differences were less robust in post-REM phases (*t* = − 2.171; *p*_Holm_ = 0.104). Moreover, the post hoc analysis showed that this significant pre- to post-REM reduction in mean HR was only a characteristic of the NM group (*t* = 4.013; *p*_Holm_ = 0.001; MD = 1.712), but not that of the CTL group (*t* = 0.649; *p*_Holm_ = 0.519) (Figs. 2a and 3a, b).

Contrasting pre- and post-REM periods, a significant main effect of group emerged regarding RMSSD (*F*(1) = 4.104; *p* = 0.048; *η*^2^_p_ = 0.076), but not HF (*F*(1) = 2.238; *p* = 0.141; η^2^_p_ = 0.043). Further post hoc analysis revealed that CTLs had higher RMSSD values than NMs (*t* = 2.026; *p*_Holm_ = 0.048; MD = 0.273; *d* = 0.281), suggesting attenuated HRV in the NM group. No significant main effects of Phase were observed in the RMSSD (*F*(1) = 2.556; *p* = 0.116; *η*^2^_p_ = 0.049) or in the HF (*F*(1) = 1.186; *p* = 0.281; *η*^2^_p_ = 0.023), and the Phase × Group interactions were not significant either (RMSSD: (*F*(1) = 0.637; *p* = 0.428; *η*^2^_p_ = 0.013; HF: (*F*(1) = 0.466; *p* = 0.498; η^2^_p_ = 0.009) (Figs. 2b, c, 3c–f).

To examine if dream anxiety or sleep quality are confounding factors that influence PA in the NM group, we carried out additional correlation analyses. The results show that none of the HR or HRV measures were significantly correlated in any of the wake or sleep states with the scores of the Van Dream Anxiety Scale or sleep quality within the NM group (see Tables S2 and S3).

### Picture-rating task

Regarding the experimental phase of the daytime picture-rating task, not only the cardiac activity was included in the analyses, but also the subjective ratings in valence and arousal.

**Fig. 4:**
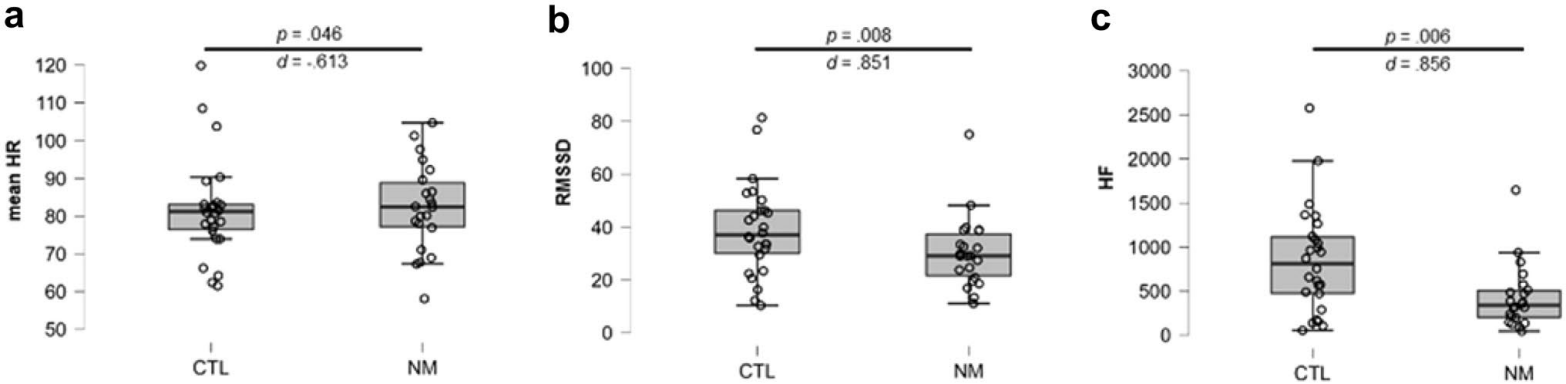
Cardiac responses (**a**–**c**) of the NM and CTL groups during the picture-rating task. *NM* nightmare group; *CTL* control group; *HR* heart rate; *RMSSD* root mean square of successive differences; *HF* high frequency component of the HRV. Error bars show confidence intervals (95%)

As for the mean HR, a significant difference was observed between the two groups, due to the higher HR of the NMs as compared to the CTLs (Fig. 4a). Furthermore, both HRV measures differentiated the two groups significantly. As Fig. 4b and c illustrate, the RMSSD and the HF indicate that the NM group had reduced HRV while encountering and rating the pictures. These physiological data suggest that NMs had a strong cardiac response in acutely arousing conditions.

Regarding the subjective ratings of the photographs, neither the valence (*t*(41) = 0.995; *p* = 0.326), nor the arousal (*t*(41) = − 0.296; *p* = 0.768) distinguished the groups significantly, which result is in contrast to the autonomic reaction of the NMs.

In sum, during the picture-rating task, the nightmare group had elevated HR and decreased HRV, whereas their subjective ratings did not significantly differ from that of the CTLs.

## Discussion

Our aim was to examine the activity of the parasympathetic nervous system in NMs as compared to a control group in order to test the hypothesis that they exhibit reduced PA during sleep and under emotional processing. It has previously been hypothesized that frequent nightmare experience could be associated with emotional dysregulation (increased emotional arousal in response to negative stimuli) and altered autonomic activity [6, 11, 14, 24, 31, 46]. Our study aimed to assess this phenomenon during different sleep and awake states. More specifically, mean HR and HRV were contrasted across the groups in different sleep states, in pre-sleep resting wakefulness and during an emotion-evoking task.

In summary, the NM group exhibited enhanced HR in all the examined sleep stages, that is, in pre-REM, REM, post-REM and stable NREM sleep, compared to CTLs. This difference emerged only in sleep and was not evidenced during resting state before sleep onset. Although the two groups did not show any differences in HRV during sleep, cardiac activity (HR, HRV) differentiated all sleep stages. As for the daytime picture-rating task, the subjective ratings of the NM and CTL groups did not differ either in valence, or in arousal; however, NMs exhibited higher HR and lower HRV during the experimental phase of the task as opposed to CTLs.

As for the physiological alterations in NMs during sleep, first of all, in resting state before sleep onset no significant differences were found between the CTLs and NMs, suggesting that the vagal tone takes over in both groups in restful state. Increased PA in resting wakefulness anticipating sleep is in line with previous observations indicating a shift from sympathetic to PA in the transition from wakefulness to sleep [38, 73, 74]. Our results indicating no differences in cardiac measures across the NM and the CTL groups before falling asleep contrast with the findings in insomnia, in which wakefulness before sleep onset is characterized by subjective pre-sleep arousal [75] and signs of reduced PA relative to controls [74, 76–78]. This contrast might imply that while hyperarousal in insomnia underlies difficulties in initiating and maintaining sleep, for nightmare recallers disrupted regulation of arousals may appear specifically during sleep [79].

The NM group exhibited significantly higher mean HR in all phases during sleep than CTLs. The elevated HR was also observed during slow wave sleep, which is generally a parasympathetically dominated sleep period [38]. With ambulatory assessment in a group of nightmare recallers and matched controls, altered HR was only found when comparing REM phases with and without nightmares [47]. Nonetheless, our result indicates that PA in NMs compared to CTLs is generally decreased beyond the state modulations of different sleep stages. This contrasts Paul and colleagues reporting altered cardiac activity on a state-level linked to the experience of dysphoric dreaming [47]. These discrepancies could be due to some important methodological differences between the two studies. While Paul and colleagues applied polysomnography in a home-based environment and concentrated on 5-min-long segments preceding awakening [47], we assessed our data in a laboratory and examined the mean values of 10-min-long REM-phase excerpts regardless of awakenings. We may speculate that the artificial, novel (and potentially somewhat stressful) environment of the laboratory might generally reduce PA in nightmare recallers compared to the home environment, where reduced PA may only emerge under periods of intense stress (i.e. nightmare episodes).

As for the HRV measures, we did not observe altered HRV in NMs during sleep. Neither HF nor RMSSD showed any significant changes across the investigated sleep stages or transitions, suggesting maintained parasympathetic tone on cardiac activity in NM participants. In order to find out the replicability of previous results based on Simor and colleagues [24], we also focused on NREM to REM and REM to NREM transitions. The mean HR had the strongest effect and showed that NMs had relatively higher HR in both pre- and post-REM. We also found reduced values of HRV in the NM group, as indexed by the RMSSD and irrespective of the sleep stage. These findings partially replicate the patterns previously observed [24], but were less consistent with respect to HRV measures. We might suppose that considering the issue of comorbidity and including participants without clinically relevant depressive and anxiety symptoms might have resulted in a sample of nightmare recallers with reduced clinical severity compared to previously examined samples [24, 46]. Furthermore, our groups were matched in dream recall rates contributing to a less biased comparison. Additionally, we used only those two HRV measures that are considered the most consistent markers of PA [53]. Additional analyses revealed nonsignificant correlations between dream anxiety or sleep quality and cardiac measures, suggesting that changes in PA in NMs during sleep are not linked to the severity of nightmare distress or reduced sleep quality.

In contrast to pre-sleep resting wakefulness, NMs exhibited signs of reduced PA during an emotionally challenging task, indicating state-specific modulations of cardiac regulation. During picture rating, NMs exhibited higher HR and reduced HRV compared to CTLs, implying decreased PA to emotional stimuli in a vigilant state. In contrast, subjective ratings in valence and arousal did not differentiate the two groups. This finding contradicts that of Carr and colleagues, whose results suggest high sensitivity of participants with frequent nightmares to images in subjective arousal, but in their work, pictures with positive valence were also presented [32]. Enhanced autonomic activity in response to an emotionally challenging task resembles the findings of Paul and colleagues, who observed attenuated HRV in nightmare recallers specifically during the experience of nightmares [47]. Altered PA while viewing emotionally arousing images in wakefulness may model the distressing oneiric experience and enhanced physiological reactivity during the occurrence of nightmares.

Although alterations in HRV throughout different sleep stages were beyond our main scope, we observed significant changes in HR and HRV between sleep periods. As sleep becomes deeper and approaches slow wave sleep, sleep is parasympathetically dominated, while in shallower phases, such as before and during REM, PA decreases [38]. Accordingly, we observed an increase in PA from pre- to post-REM (regardless of the group) as well as the reduction of parasympathetic influence from NREM to REM.

Our findings indicating altered physiological activity in NMs not only during sleep, but also unequivocally in response to emotional stimuli are in line with the predictions of the ‘affect load’ element of the neurocognitive model of nightmares [6], as well as more recent theories expressed by Nielsen [11] and Ellis [14]. Nielsen and Levin emphasize the link between daytime psychological functioning and nocturnal disturbances in nightmare disorder, due to potentially overlapping neural activity in emotion processing networks during wakefulness and dreaming [6, 31]. Our assumption that chronic nightmare recallers exhibit altered cardiac activity reflected in HR and HRV irrespective of nightmare presence was partially supported (‘affect distress’ component [6]). It should be noted, however, that our sample was free of comorbidities and hence, may represent the non-pathological side of the spectrum of dysphoric dreaming [9]. Frequent nightmares associated with distress and trauma may be considered more severe pathological states [9]. Accordingly, frequent nightmares appear as severe, debilitating symptoms in PTSD [80]. Furthermore, PTSD patients exhibit reduced vagal tone in daytime resting states [45] and also during sleep [44]. Taking these results into account, more severe symptoms associated with nightmares, such as in PTSD, might lead to more consistent cardiac responses, and thus to more dysregulated PA during sleep.

Important limitations of our study include the absence of the evaluation of the sympathetic activity, which would be possible with other measurements, such as impedance cardiography [81]. Additionally, we had a generally healthy sample consisting of mostly university students, not patients with clinically severe suffering. Finally, due to the controlled conditions of a laboratory setting, the results are less ecologically valid, since sleeping away from home might have had an impact on participants’ autonomic activity.

## Conclusion

To conclude, in this study we attempted to gain a better knowledge of the trait- and state-like autonomic alterations of chronic nightmare recallers relative to healthy controls. In order to understand these differences in greater depth, further studies are needed that differentiate the severity of the nightmare experience when evaluating their autonomic activity. Home-based experiments are also necessary, in which prospective ambulatory assessment and subjective data collection would enable researchers to gather an in-depth knowledge of the characteristics of frequent idiopathic nightmares on trait- and state-levels.

### Supplementary Information

Below is the link to the electronic supplementary material.

Supplementary file1 (DOCX 105 kb)

## Data Availability

The data included in this study are available here: https://osf.io/jg82m/?view_only=125cf6b6afeb4e64bb532124caca1f68

## Material availability

The materials applied in this study are available here:https://osf.io/jg82m/?view_only=125cf6b6afeb4e64bb532124caca1f68
