# Supplementary material for: Altered parasympathetic activity during sleep and emotionally arousing wakefulness in frequent nightmare recallers
Source: Eur Arch Psychiatry Clin Neurosci. 2023 Mar 2;274(2):265–77. doi: 10.1007/s00406-023-01573-2 (PMC10914885; doi:10.1007/s00406-023-01573-2)
Supplement: Supplementary file 1 — Supplementary file1 (DOCX 105 kb) [file 406_2023_1573_MOESM1_ESM.docx]

**Altered parasympathetic activity during sleep and emotionally arousing wakefulness in frequent nightmare recallers**

**– Supplementary Material –**

Vivien Tomacsek^1,2^, Borbála Blaskovich^3^, Anna Király^4^, Richárd Reichardt^2,5^, Péter Simor^2,6^

1 *Doctoral School of Psychology, ELTE Eötvös Loránd University, Budapest, Hungary.*

2 *Institute of Psychology, ELTE Eötvös Loránd University, Budapest, Hungary.*

3 *Institute of Medical Psychology, Faculty of Medicine, Ludwig Maximilian University of Munich, Munich, Germany.*

4 *National Institute of Locomotor Diseases and Disabilities, Budapest, Hungary.*

5 *Department of Cognitive Science, Budapest University of Technology and Economics, Budapest, Hungary.*

6 *UR2NF, Neuropsychology and Functional Neuroimaging Research Unit at CRCN - Center for Research in Cognition and Neurosciences and UNI - ULB Neurosciences Institute, Université Libre de Bruxelles (ULB), Brussels, Belgium.*

*Corresponding author:*

Vivien Tomacsek

*Institute of Psychology, ELTE Eötvös Loránd University, Budapest, Hungary.*

1064 Budapest, Izabella Street 46.

[tomacsek.vivien@ppk.elte.hu](mailto:tomacsek.vivien@ppk.elte.hu)

+36209786203

| **Table S1.**  *Descriptive statistics based on the dream anxiety, depression and trait anxiety scores* | | | | | | |
| --- | --- | --- | --- | --- | --- | --- |
|  | **VDAS** | | **BDI** | | **STAIT-T** | |
|  | **CTL** | **NM** | **CTL** | **NM** | **CTL** | **NM** |
| **Valid** | 30 | 24 | 30 | 24 | 30 | 23 |
| **Mean** | 2.033 | 18.333 | 3.167 | 6.458 | 41.733 | 47.826 |
| **Standard Deviation** | 2.484 | 9.164 | 2.394 | 6.311 | 8.179 | 13.466 |
| **Minimum** | 0 | 5 | 0 | 0 | 21 | 30 |
| **Maximum** | 9 | 39 | 9 | 22 | 57 | 71 |
| *Note.* VDAS = Van Dream Anxiety Scale; BDI = Beck’s Depression Inventory; STAIT-T = State-Trait Anxiety Inventory – Trait items. CTL = control participants; NM = frequent nightmare recallers. | | | | | | |

| **Table S2.**  *Correlations between dream anxiety and HR and HRV variables within the NM group* | | | | | | | | | | | | | | | | | | |
| --- | --- | --- | --- | --- | --- | --- | --- | --- | --- | --- | --- | --- | --- | --- | --- | --- | --- | --- |
|  | | **MEAN HR** | | | | | **RMSSD** | | | | | | **HF** | | | | | |
|  |  | **pre-REM** | **post-REM** | **stable NREM** | **REM** | **wake** | **pre-REM** | **post-REM** | **stable NREM** | **REM** | **wake** | **pre-REM** | | **post-REM** | **stable NREM** | **REM** | **wake** |  |
| **V**  **D**  **A**  **S** | Correla-tion coeffici-ent | .262 | .198 | .117 | .230 | -.091 | -.063 | -.020 | .011 | -.080 | .236 | -.108 | | -.017 | -.007 | -.112 | .203 |  |
|  | *p*-value | .265 | .403 | .624 | .330 | .703 | .791 | .933 | .964 | .737 | .316 | .651 | | .945 | .976 | .639 | .390 |  |
| *Note.* Bootstrap results are based on 1000 bootstrap samples. The correlation coefficient is Pearson’s. HR = heart rate; HRV = heart rate variability, NM = frequent nightmare recaller. VDAS = Van Dream Anxiety Scale; RMSSD = root mean square of successive differences; HF = high frequency component of the HRV; REM = rapid eye movement; NREM = non-rapid eye movement. | | | | | | | | | | | | | | | | | | |

To find out whether the changes in frequent nightmare recallers in parasympathetic activity can be attributed to reduced sleep quality, some additional correlation analyses were performed. The participants reported on their subjective sleep quality in the morning using a single item assessing sleep quality. The item had to be rated on a 9-point Likert scale based on how they evaluated their sleep quality the previous night (1: *I slept very poorly*; 9: *I slept very well and soundly*). In addition to the self-reported sleep quality, using the polysomnographic (PSG) data, we were able to extract the participants’ sleep efficiency. This way, it was possible to examine the relationship between the heart rate (HR) or heart rate variability (HRV) variables and a subjective and a physiological measure of sleep quality. To better understand the impact of subjective sleep quality and sleep efficiency on the HRV measures, we performed correlation analyses in the two groups (i.e. frequent nightmare recallers – NM and healthy control participants – CTL) separately; see **Tables S3** and S**4** for the results, respectively.

| **Table S3.**  *Correlations between sleep quality and sleep efficiency, and HR and HRV variables within the NM group* | | | | | | | | | | | | | | | | | |
| --- | --- | --- | --- | --- | --- | --- | --- | --- | --- | --- | --- | --- | --- | --- | --- | --- | --- |
|  | | **MEAN HR** | | | | | **RMSSD** | | | | | **HF** | | | | | |
|  |  | **pre-REM** | **post-REM** | **stable NREM** | **REM** | **wake** | **pre-REM** | **post-REM** | **stable NREM** | **REM** | **wake** | **pre-REM** | **post-REM** | **stable NREM** | **REM** | **wake** |  |
| **Subjective sleep quality** | Corre-lation coeffi-cient | .115 | .291 | .315 | .265 | .219 | -.133 | -.331 | -.277 | -.303 | -.210 | .029 | -.249 | -.129 | -.199 | -.096 |  |
|  | *p*-value | .628 | .214 | .176 | .259 | .353 | .576 | .154 | .238 | .194 | .373 | .905 | .291 | .587 | .401 | .686 |  |
| **Sleep efficiency (PSG measure)** | Corre-lation coeffi-cient | -.140 | -.237 | -.288 | -.155 | -.397 | .046 | .099 | .154 | .149 | .228 | .029 | .115 | .146 | .121 | .131 |  |
|  | *p*-value | .557 | .314 | .218 | .513 | .083 | .846 | .678 | .517 | .532 | .333 | .904 | .630 | .540 | .610 | .583 |  |
| *Note.* Bootstrap results are based on 1000 bootstrap samples. The correlation coefficient is Pearson’s. HR = heart rate; HRV = heart rate variability; NM = frequent nightmare recaller. PSG = polysomnographic; RMSSD = root mean square of successive differences; HF = high frequency component of the HRV; REM = rapid eye movement; NREM = non-rapid eye movement. | | | | | | | | | | | | | | | | | |

| **Table S4.**  *Correlations between sleep quality and sleep efficiency, and HR and HRV variables within the CTL group* | | | | | | | | | | | | | | | | | |
| --- | --- | --- | --- | --- | --- | --- | --- | --- | --- | --- | --- | --- | --- | --- | --- | --- | --- |
|  | | **MEAN HR** | | | | | **RMSSD** | | | | | **HF** | | | | | |
|  |  | **pre-REM** | **post-REM** | **stable NREM** | **REM** | **wake** | **pre-REM** | **post-REM** | **stable NREM** | **REM** | **wake** | **pre-REM** | **post-REM** | **stable NREM** | **REM** | **wake** |  |
| **Subjective sleep quality** | Corre-lation coeffi-cient | .141 | .078 | .057 | .120 | .097 | -.177 | -.212 | -.205 | -.172 | -.140 | -.201 | -.228 | -.249 | -.213 | -.155 |  |
|  | *p*-value | .474 | .693 | .774 | .542 | .624 | .368 | .279 | .296 | .381 | .477 | .304 | .243 | .201 | .277 | .432 |  |
| **Sleep efficiency (PSG measure)** | Corre-lation coeffi-cient | -.020 | -.086 | -.080 | -.091 | -.266 | -.052 | -.036 | -.002 | .031 | .261 | -.103 | -.121 | -.071 | -.015 | .211 |  |
|  | *p*-value | .918 | .663 | .686 | .644 | .171 | .792 | .857 | .993 | .876 | .180 | .601 | .538 | .718 | .939 | .281 |  |
| *Note.* Bootstrap results are based on 1000 bootstrap samples. The correlation coefficient is Pearson’s. HR = heart rate; HRV = heart rate variability; CTL = control. PSG = polysomnographic; RMSSD = root mean square of successive differences; HF = high frequency component of the HRV; REM = rapid eye movement; NREM = non-rapid eye movement. | | | | | | | | | | | | | | | | | |

The participants might have had a nightmare or bad dream on the study night. To see if they did, we examined their answer to a question that they could answer in the morning questionnaire if they were able to spontaneously recall their dreams and/or emotions. The item assessed the emotional tone of their dreams (*The affective load of the dream was… (0) particularly unpleasant, (1) unpleasant, (2) neutral, (3) pleasant, (4) particularly pleasant*). The answers (0) and (1) could be equal to having a nightmare or bad dream. **Figure S1** shows that participants in the NM group had lower scores on the item, referring to the fact that the emotional quality of their dreams was more negative than that of the CTLs.


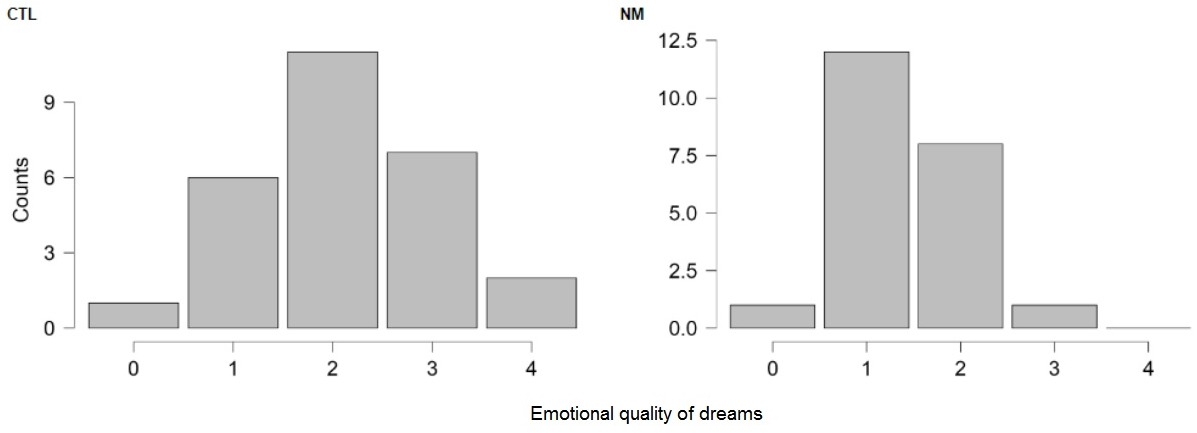


**Figure S1.** The scores of the control and nightmare groups’ evaluation on the emotional quality of their dreams.

To further explore this association, we performed correlation analyses with emotional quality of the NMs and the HR and HRV variables. As you can see in **Table S5** below, the emotional quality did not correlate with any of the HR or HRV measures significantly, reinforcing our hypothesis that altered parasympathetic activity does occur in NMs regardless of a current nightmare episode.

| **Table S5.**  *Correlations between the emotional quality of dreams and HR and HRV variables within the NM group* | | | | | | | | | | | | | | | | | |
| --- | --- | --- | --- | --- | --- | --- | --- | --- | --- | --- | --- | --- | --- | --- | --- | --- | --- |
|  | | **MEAN HR** | | | | | **RMSSD** | | | | | **HF** | | | | | |
|  | | **pre-REM** | **post-REM** | **stable NREM** | **REM** | **wake** | **pre-REM** | **post-REM** | **stable NREM** | **REM** | **wake** | **pre-REM** | **post-REM** | **stable NREM** | **REM** | **wake** |  |
| **Emotional quality of dreams** | Corre-lation coeffi-cient | .084 | .222 | .255 | .104 | -.128 | -.084 | -.211 | -.241 | -.095 | .158 | .013 | -.173 | -.125 | -.029 | .156 |  |
|  | *p*-value | .739 | .376 | .307 | .680 | .613 | .741 | .400 | .336 | .709 | .531 | .959 | .492 | .621 | .910 | .537 |  |
| *Note.* Bootstrap results are based on 1000 bootstrap samples. The correlation coefficient is Pearson’s. HR = heart rate; HRV = heart rate variability; NM = frequent nightmare recaller. RMSSD = root mean square of successive differences; HF = high frequency component of the HRV; REM = rapid eye movement; NREM = non-rapid eye movement. | | | | | | | | | | | | | | | | | |
